# NO_x_-Reduction Performance Test for TiO_2_ Paint

**DOI:** 10.3390/molecules25184087

**Published:** 2020-09-07

**Authors:** Yong Woo Song, Min Young Kim, Min Hee Chung, Young Kwon Yang, Jin Chul Park

**Affiliations:** 1 Graduate School, Chung-Ang University, Seoul 06794, Korea; yongma0930@cau.ac.kr; 2 School of Architecture and Building Science, Chung-Ang University, Seoul 06974, Korea; kmyhg@naver.com (M.Y.K.); mhloveu@cau.ac.kr (M.H.C.); dora84@naver.com (Y.K.Y.)

**Keywords:** titanium dioxide (TiO_2_), nitrogen oxides (NO_x_), particulate matter, secondary source, reduction test

## Abstract

In South Korea, the gradual increase in particulate matter generation has received significant attention from central and local governments. Exhaust gas, which contains nitrogen oxides (NO_x_), is one of the main sources of particulate matter. In this study, the reduction of NO_x_ using a coating material mixed with a titanium dioxide (TiO_2_) photocatalyst was demonstrated. The NO_x_ reduction performance of the TiO_2_ photocatalyst-infused coating was evaluated by applying the ISO 22197-1: 2007 standard. Subsequently, the performance was evaluated by changing the NO gas concentration and ultraviolet (UV)-A irradiance under standard experimental conditions. It was determined that NO_x_ reduction can be achieved even if the NO gas concentration and UV-A irradiance are lower than those under the standard conditions when the TiO_2_ photocatalyst-infused coating was used. This study revealed that NO_x_ reduction can be realized through TiO_2_ photocatalyst-infused coating in winter or cloudy days with a low solar altitude. It was also confirmed that compared with the UV-A irradiance, the NO gas concentration has a greater effect on the NO_x_ reduction performance of the TiO_2_ photocatalyst-infused coating. These findings can be used to evaluate a variety of construction materials with TiO_2_ photocatalysts in the future.

## 1. Introduction

Since the gradual increase in particulate matter (PM) concentration has been recognized as a national problem in South Korea, central and local governments have made considerable efforts to reduce such concentration. According to data from the World Health Organization (WHO), the annual average PM concentration of Seoul, South Korea in 2016 was 46 µg/m^3^, which is 1.2–3.5 times higher compared with Tokyo, Japan (28 µg/m^3^), Paris, 1‘France (28 µg/m^3^), and Washington DC, USA (16 µg/m^3^) [1].

The causes of PM are divided into primary and secondary sources. Primary sources are pollutants that are directly generated by pollution sources, and their particle sizes are less than 10 µm (PM_10_). The introduction of primary sources into indoor spaces can be reduced by applying high-efficiency particulate air (HEPA) filters to indoor air purifiers and heating, ventilating, and air-conditioning (HVAC) systems [2,3,4].

Secondary sources are generated through chemical bonding between gaseous pollutants in primary pollutants and precursors in the atmosphere, and their particle sizes are generally less than 2.5 µm (PM_2.5_). These pollutants vary in size, weight, color, and their internal components. Their main source is the exhaust gas that is generated through transportation, which includes automobiles. This is due to the incomplete combustion of fossil fuels; thus, their concentrations are high in downtown areas with large traffic volumes [5]. Sulfur oxides (SOx) and nitrogen oxides (NO_x_) account for approximately 58% of all substances causing secondary sources, which is the highest proportion [6].

In particular, the representative substance that generates secondary sources in South Korea is NO_x_, which represents 42.3% of all substances that can cause secondary sources [7,8]. Therefore, it is expected that NO_x_ reduction will decrease the overall PM concentration in South Korea.

The representative materials that can reduce secondary sources through chemical reactions are TiO_2_ photocatalysts. They can achieve self-cleaning [9,10,11,12,13,14,15,16], antibacterial resistance [16], air-cleaning, deodorization [16,17,18,19,20,21,22,23], and water-purification [23,24,25,26,27] effects by absorbing light in the 350–380 nm range of the wavelengths of light. Among them, air cleaning was used in this study because it can remove the cause of PM generation by oxidizing NO_x_. This can reduce PM through photochemical reaction with ultraviolet (UV) rays. The photochemical reaction between TiO_2_ and UV rays is given in Equation (1) [28]. The photocatalytic oxidation mechanism of nitrogen oxides is given by

(1) $$\mathrm{Activation}:\;{\mathrm{TiO}}_{2}+{}^{*}\mathrm{hv}\to h^{+}+e^{-}$$

(2) $$H_{2}O\left(g\right)+{\mathrm{Site}}^{**}\to H_{2}O_{\mathrm{ads}}$$

(3) $$O_{2}\left(g\right)+{\mathrm{Site}}^{**}\to O_{2\mathrm{ads}}$$

(4) $$\mathrm{NO}\left(g\right)+{\mathrm{Site}}^{**}\to {\mathrm{NO}}_{\mathrm{ads}}$$

(5) $${\mathrm{NO}}_{2}\left(g\right)+{\mathrm{Site}}^{**}\to {\mathrm{NO}}_{2\mathrm{ads}}$$

(6) $$\mathrm{Hole}\;\mathrm{trapping}:\;H_{2}O+h^{+}\to \;•\mathrm{OH}+H^{+}$$

(7) $$\mathrm{Electron}\;\mathrm{trapping}-:\;O_{2}\left(g\right)+e^{-}\to O_{2}{}^{-}$$

(8) $$\mathrm{Hydroxyl}\;\mathrm{attack}:\;{\mathrm{NO}}_{\mathrm{ads}}+2•\mathrm{OH}\to {\mathrm{NO}}_{2\mathrm{ads}}+H_{2}O\;\;{\mathrm{NO}}_{2\mathrm{ads}}+•\mathrm{OH}\to {\mathrm{HNO}}_{3}$$

*hv: light (ultraviolet radiation), **Site: surface of TiO_2_, •OH: hydroxyl radical

As shown in Equations (1)–(8), when TiO_2_ on the material surface absorbs energy from UV rays in sunlight, holes (h^+^) and electrons (e^−^) are generated (Equation (1)). •OH and O_2_^−^ • radicals with a strong oxidizing power are generated on the surface of TiO_2_ through reactions with H_2_O and O_2_ in the atmosphere (Equations (2)–(7), electron trapping).

This can lead to the hydroxyl attack step (Equation (8)) in which NO_x_, a representative precursor, is decomposed [29,30]. Through this mechanism, secondary sources such as NO_x_ are decomposed on the photocatalyst surface, resulting in PM reduction effect. With the development of light source technology, concrete and cement mixed with TiO_2_ photocatalysts have recently been actively developed. From 2000 to 2010, only two studies were conducted in which TiO_2_ photocatalysts were mixed with concrete and cement [31,32]. However, seven studies were conducted from 2010 to 2020 [29,33,34,35,36,37,38]. These studies mainly focused on road pavement materials, exterior materials, and roof finishing materials.

A review of previous studies on the application of TiO_2_ is presented below. The most representative applications are cement road materials, which include pavement blocks [29], asphalt pavement [33,36,37], and mortar [34,35]. The reduction in NO_x_ concentration was examined by adding TiO_2_ photocatalysts to these cement road materials, and it was observed that the NO_x_ concentration can be reduced by up to 60–80% [31]. Yu et al. investigated NOx concentration reduction performance based on automobile exhaust gas concentration through field measurement and verified the optimal mass ratio [36]. Wang et al. investigated the effect of exhaust gas decomposition by setting the UV irradiance as 26.7 W/m^2^, which is the average outdoor illumination, and it was confirmed that exhaust gas decomposition can be achieved when the nano-TiO_2_ content is 8% [37]. Witkowski et al. conducted NO removal experiment using a photocatalytic pavement block used for 7 years on a silver bicycle road, and the NO gas reduction performance was investigated by applying 300 W of light; scanning electron microscopy (SEM) and energy dispersive spectroscopy (EDS) mapping analyses were performed to confirm TiO_2_ mixing in the pavement block [38]. The irradiance of UV applied in the experiment was very high compared with the atmosphere; therefore, the actual environment should be considered while conducting the experiment.

Cassar et al. conducted a study on the application of TiO_2_ photocatalysts to build an exterior other than road pavement. As a representative case, the Italcementi group in Italy installed an exterior material mixed with TiO_2_ photocatalysts in the Roman Jubilee Cathedral and road pavements on a trial basis. They confirmed an antifouling performance and air pollution reduction of 30–40% [32]. 

In addition, Luna et al. applied the TiO_2_ photocatalyst coating to limestone and granite surfaces. They found that granite is better than limestone in terms of its antifouling performance and smoke removal [39]. Lettieri et al. applied a TiO_2_ photocatalyst coating to a limestone surface and investigated its NO_x_ reduction performance. They also found that the antifouling performance was lost after 8 months [40].

Ramirez et al. applied a TiO_2_ photocatalyst coating and particle injection to porous cementitious materials. They determined that the use of the particle injection method on porous materials results in a high toluene removal efficiency [20]. Tang et al. coated TiO_2_ photocatalysts in the form of granules on building roofs in addition to the exterior materials to investigate their self-cleaning characteristics. They found that these photocatalysts were effective, even for the urban heat island phenomenon, owing to the observed lower surface temperatures [41].

As described above, most recent studies on TiO_2_ focused on the evaluation of air-cleaning performance using cement and stone. In addition, a few studies have been conducted on the quantitative evaluation of the air-cleaning performance of TiO_2_ combined with other building materials.

In this study, two tests were conducted to examine the air-cleaning performance of building materials that utilized the photochemical properties of TiO_2_ photocatalysts (activation in the 350–380 nm range). The building material used in the tests was a coating material mixed with TiO_2_ photocatalysts; this was utilized in this study because it can be applied to a variety of building materials or the structures of existing buildings.

In the first test, the ISO 22197-1:2007 test method was used with the coating material mixed with TiO_2_ photocatalysts to examine its NO_x_ reduction performance in secondary sources [42]. The second test was conducted to examine the quantitative performance change of TiO_2_ photocatalysts when the NO concentration and UV-A irradiance based on the ISO 22197-1:2007 test conditions were changed. The results of each test are analyzed in Section 2, and they are combined and used to analyze the NO_x_ reduction performance of TiO_2_ photocatalysts as presented in Section 3. It is expected that the results of this study can be used to reduce PM concentrations in buildings in the future.

## 2. Materials and Methods

### 2.1. Materials and Experimental Overview

In this study, a TiO_2_ coating material was used to evaluate its NO_x_ reduction performance. To this end, the TiO_2_ coating material (ZT-01 from Bentech Frontier, Jeollanam-do, South Korea) was applied on a Pyrex glass specimen along with a primer. The primer was applied before the coating to make the back surface of the specimen opaque and block external light that may enter the back surface. 

The TiO_2_ coating material was made of anatase-based titanium dioxide. The detailed physical properties are listed in Table 1. 

**Figure 1:**
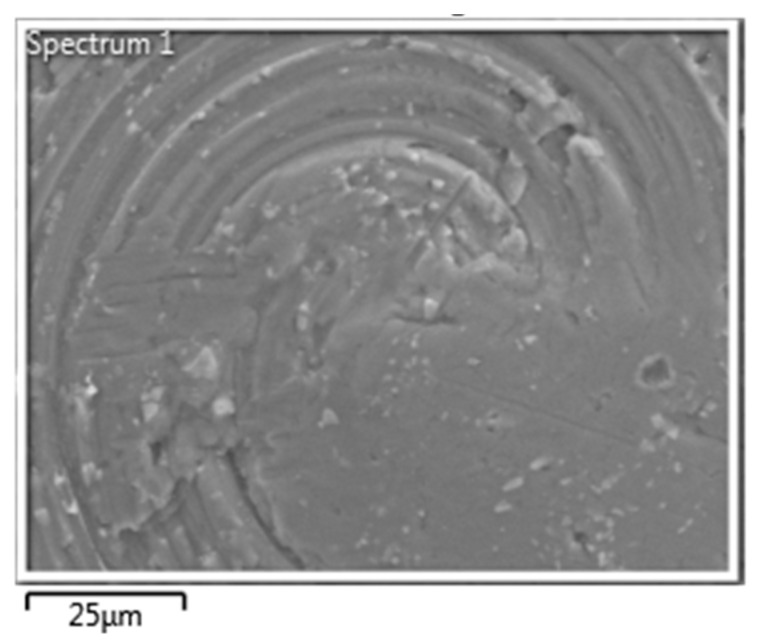
STUB (Sample Holder) field emission scanning electronic microscope (FESEM) image.

FESEM (field emission scanning electronic microscope, Gwangju, Republic of Korea) and EDS mapping analyses were (Gwangju, Republic of Korea) performed on the ZT-01 material used in the experiment. The contents of the equipment used for FESEM and EDS analysis are as in Table 2. The analyses were performed on pre- and postcoating STUB (sample holder)s, and the STUBs before and after the application were analyzed. The materials in the STUB before the application of TiO_2_ coating material were composed of C (12.84%), O (3.04%, Al (79.34%), and Cu (4.79%) (See Table 3, Figure 1).

**Figure 2:**
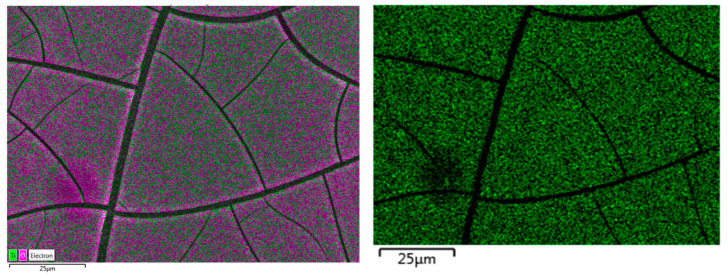
TiO_2_ coating FESEM and energy dispersive spectroscopy (EDS) mapping image.

After applying the TiO_2_ coating material to the STUB, FESEM, and EDS mapping analyses showed that it was the same as Table 4 and Figure 2, and TiO_2_ was included in coating material approximately 21.05%.

Through the analysis of FESEM and EDS mappings, sufficient Ti and O components were present inside the TiO_2_ coating agent used in this experiment to determine their effect on the reduction of NO_x_.

The size of the specimen that was used (10 cm^2^) was small; hence, the TiO_2_ coating was applied using a brush. The amount of coating used was calculated from the precoating brush weight, the postcoating brush weight, the precoating specimen weight, and the postcoating specimen weight difference; approximately 12 g of coating was applied. In this study, we focused on measuring the volume of coating rather than the coating thickness.

Coating was applied one to 10 times, and preliminary experiments were conducted to confirm the NOx reduction in efficiency. This study confirmed that the same efficiency can be achieved seven times. After applying the coating, the specimen surface was confirmed to be cured and smooth. 

The size of the prepared specimen was 10 mm × 100 mm (width × height), which is based on the flow rate of 3 lpm specified in the ISO 22197-1:2007.

The NO_x_ reduction performance test was conducted using the following two methods. First, it was conducted by applying NO 1.00 ppm and UV-A 10 W/m^2^, which are the standard conditions in the ISO 22197-1:2007. Second, changes in the NO_x_ concentration were measured, while the NO gas and UV-A concentrations were reduced at a constant rate. The UV-A irradiance was reduced by adjusting the vertical distance between the specimen and the lamp. The UV-A lamps used in this experiment consisted of TL-D 18 W BL lamps (P Company), irradiating light from 315–400 nm. The UV-A includes the active wavelengths of the TiO_2_ photocatalysts, and it represents 95% of the UV rays that reach the ground surface [43]. The interexperimental TiO_2_ photocatalyst coating specimen was used with distilled water for cleaning.

**Figure 3:**
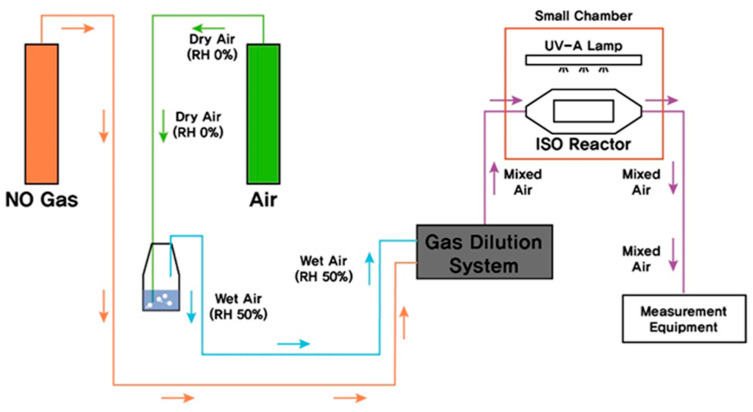
Test chamber diagram.

To measure the temperature and humidity, we used KIMO’s multipurpose measuring instrument with a SOM 900 probe. Serinus 40 (Ecotech’s NO_x_ Gas Analyzer) was used to measure the NO, NO_2_, and NO_x_ concentrations (Figure 3 and Table 5).

### 2.2. Experimental Methods on NO_x_ Reduction Using Titanium Oxide Photocatalyst

In the ISO 22197-1 standard condition test, changes in the NO, NO_2_, and NO_x_ concentrations were measured according to the on/off status of the UV-A lamp, whereas the NO 1.00 ppm and UV-A 10 W/m^2^ conditions in the chamber were continuously maintained for 3 h. The average values of three measurements were used to reduce the errors.

The UV-A irradiance used in the test was 10 W/m^2^. This is similar to the annual winter level (13.4 W/m^2^) UV irradiance in South Korea according to the statistics of the comprehensive climate change monitoring information from the Korea Meteorological Administration (KMA) [44]. The statistics from KMA, however, were values measured on horizontal surfaces. These values are expected to be lower when the actual TiO_2_ photocatalysts are utilized owing to vertical surfaces and other obstacles.

The changes in the NO, NO_2_, and NO_x_ concentrations measured in the test were analyzed using the amount of NO reduced (a) and the amount of NO_2_ generated (b) according to the on/off status of the UV-A lamp to calculate the amount of NO_x_ reduced (a − b). Table 6 lists the detailed measurement conditions.

### 2.3. Experimental Methods Based on the UV-A Irradiance and Changes in the Concentration of NO

The test for changes in the condition was conducted to examine the changes in the NO_x_ reduction performance of the TiO_2_ photocatalysts by varying the UV-A irradiance and the NO concentration.

The irradiance was set to 7.50 W/m^2^ for 25% cloud cover, 5.00 W/m^2^ for 50% cloud cover, and 2.50 W/m^2^ for 75% cloud cover. This was to simulate low cloud cover situations in winter based on the ISO standard of 10 W/m^2^. In this experiment, changes in the NO_x_ concentration were measured. 

In addition, changes in the NO_x_ concentration were measured when the NO concentration was reduced by 25%, 50%, and 75%, which are 0.75, 0.50, and 0.25 ppm, respectively, according to the ISO standard of 1.00 ppm (Table 7).

The amount of NO reduced (a), the amount of NO_2_ generated (b), and the amount of NO_x_ reduced (a − b) were calculated using the measurement results. For the conditions other than the irradiance and concentration, the standard conditions were applied.

## 3. Results and Discussion

### 3.1. Reactivity at ISO 22197-1 Standard Condition

**Figure 4:**
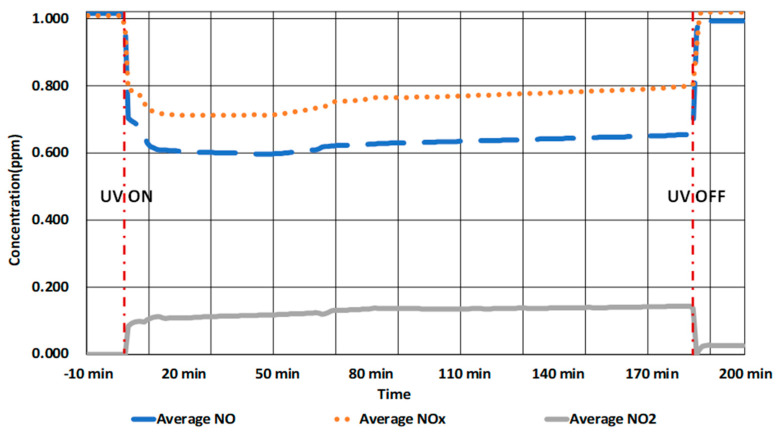
NO_x_ reduction results for the ISO standard condition.

The results of the test that applied the ISO 22197-1:2007 standard conditions (1.00 ppm and 10.0 W/m^2^) are presented in Table 8 and Figure 4. In the test, changes in the NO_x_ concentration were investigated according to the UV-A on/off status after maintaining the standard condition concentration of 1.00 ppm in the chamber.

As shown in Figure 4, the NO_x_ concentration reduced from 1.00 ppm to approximately 0.8 ppm when the UV lamp was turned on. However, it returned to 1.00 ppm when the lamp was turned off. This confirmed that the operation of the UV lamp reduces the NO_x_ concentration.

It can be observed from Table 8 that the NO concentration decreased by 35.67% (12.06 µmol/10 cm^2^·3 h) and the NO_x_ concentration by 21.03% (8.95 µmol/10 cm^2^·3 h) in 3 h, which confirms the NO_x_ concentration reduction performance of the TiO_2_ coating material. μmol/10 cm^2^·3 h is a unit using which the concentration value measured in parts per million is expressed as a quantity. The 10 cm^2^·3 h expressed behind the unit represents 10 cm^2^, the size of the experimental specimen, and 3 h, the experimental time.

In addition, it can be observed from Figure 4 and Table 8 that NO_2_ was generated when the UV lamp was turned on and, approximately, 3.11 µmol/10 cm^2^·3 h was generated until the lamp was turned off. This, in addition to the difference in the NO_x_ concentration, confirms the occurrence of oxidation reactions through the combination of TiO_2_ photocatalysts and UV rays.

### 3.2. Reactivity at Different UV-A and NO Concentrations

Although the above test was conducted by applying the ISO standard conditions (UV-A: 10 W/m^2^, NO: 1.00 ppm), the test to evaluate changes in the NO_x_ concentration was conducted by changing the UV-A irradiance and NO concentration. 

**Figure 5:**
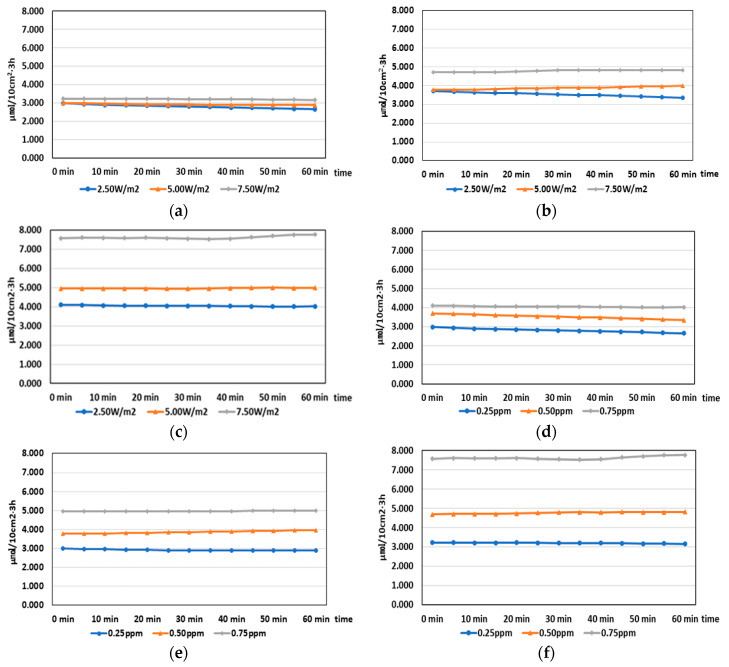
Test results of changes in condition to achieve NO_x_ reduction. NO_x_ reduction according to change in the irradiance based on a concentration of (**a**) 0.25 ppm, (**b**) 0.50 ppm, and (**c**) 0.75 ppm. NO_x_ reduction according to the change in the reference concentration at a light rate of (**d**) 2.50 W/cm^2^, (**e**) 5.00 W/cm^2^, and (**f**) 7.50 W/cm^2^.

The test results are presented in Table 9, Table 10, and Table 11 and Figure 5. This test was also conducted according to the on/off status of the UV-A lamp while a certain concentration was maintained in the chamber. 

Table 9 presents the NO reduction results according to the UV-A irradiance and NO concentration. The applied UV-A irradiance ranged from 2.5 to 7.5 W/m^2^, and the applied NO concentration ranged from 0.25 to 0.75 ppm. It was determined that the NO concentration reduction increased as the NO concentration increased under a constant UV-A irradiance. This is because the molecular weight of NO increased as the NO concentration increased when the UV-A wavelength energy that affects the TiO_2_ photocatalysts is the same.

Under a constant NO concentration, however, there was no significant trend in the cases of 0.25 and 0.50 ppm as the irradiance increased. Meanwhile, the 0.75 ppm case showed an increase in NO concentration reduction with increasing irradiance. This result shows that the NO reduction effect can be obtained by the TiO_2_ photocatalyst at concentrations of 0.5 ppm or less; however, the effect is not proportional to the amount of light. The above results indicate that the NO concentration affects the NO reduction rather than the UV-A irradiance.

Table 10 presents the NO_2_ generation results according to the NO concentration and UV-A irradiance. NO_2_ is an indicator of oxidation reactions because it is generated through photochemical reactions between UV-A and TiO_2_ photocatalysts. The NO_2_ generation results confirmed that there were photochemical reactions between UV-A and TiO_2_ photocatalysts during the test, with changes in the UV-A irradiance and NO concentration. However, NO_2_ was generated through a combination with NO and oxygen in the atmosphere. Thus, the amount of NO_2_ generated did not exhibit a constant trend with an increase in the UV-A irradiance and NO concentration.

Table 11 presents the NO_x_ reduction results according to the NO concentration and UV-A irradiance. When the UV-A irradiance was changed under the same NO concentration, the NO_x_ concentration reduction increased in proportion to the reduction rate. This is because the amount of •OH and O_2_^−^ • radicals that were generated on the surface of the coating material changed according to the UV-A irradiance, thereby reducing the amount of oxidized NO_x_. Figure 5a–c show the results according to changes in the corresponding conditions. These were calculated for 1 h after stabilization of the concentration reduction. The results confirmed that the concentration reduction varied depending on the irradiance.

In addition, when the NO concentration was changed under the same UV-A irradiance, the NO_x_ concentration reduction increased as the NO concentration increased. However, at 0.25 and 0.5 ppm, which are low NO concentrations, there is a difference in the reduction amount according to the change in the light amount for 60 min. As the concentration decreased, the difference became less. Figure 5d–f show the results of the corresponding tests. The results confirm that there were certain differences in the concentration reduction as the NO concentration changed under the same irradiance.

## 4. Discussion

**Figure 6:**
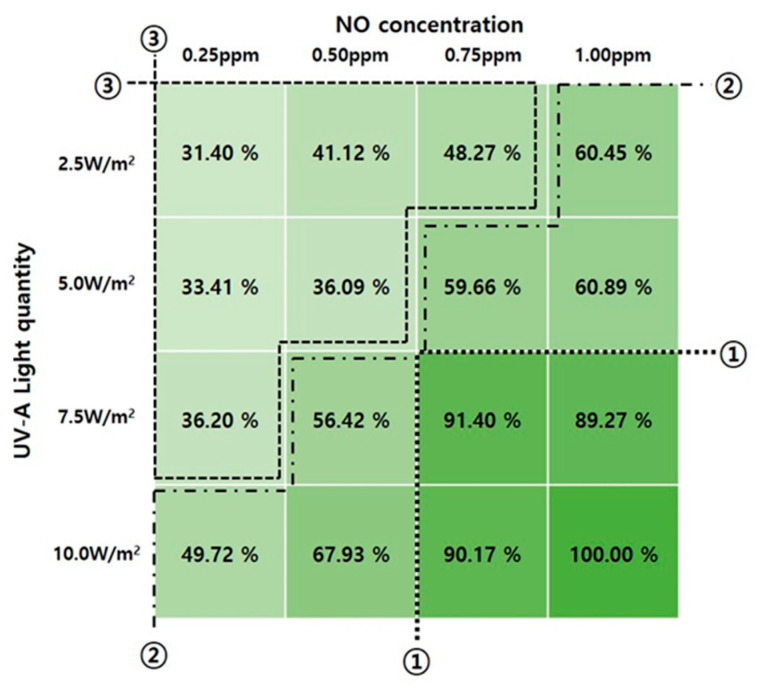
Results of NO_x_ reduction rate.

**Figure 7:**
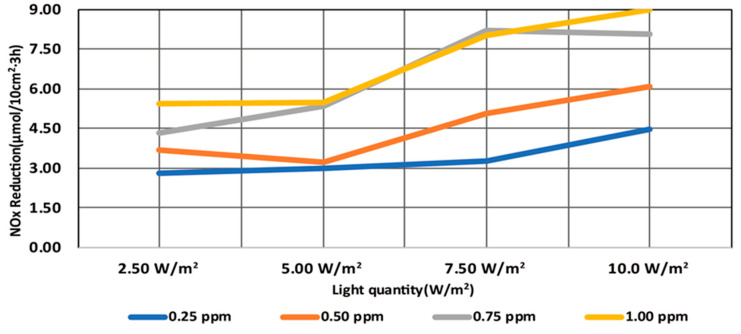
Total NO_x_ reduction test results.

The NO_x_ reduction performance test of the TiO_2_ photocatalysts was conducted using the photocatalyst coating material. The test was conducted by applying the ISO standard conditions and changing the irradiance and NO concentration. The test results are presented in Table 12, Figure 6, and Figure 7.

According to the results of the test under the standard conditions according to the ISO 22197-1:2007 (NO concentration: 1.00 ppm, UV-A irradiance: 10 W/m^2^), the amount of NO_x_ that was reduced was 8.95 µmol/10 cm^2^·3 h, as presented in Table 12 and Figure 7. In addition, when the NO concentration and UV-A irradiance were low, the amount of NO_x_ that was reduced ranged from 2.81 to 8.18 µmol/10 cm^2^·3 h.

Moreover, when the reduction amount confirmed that the standard condition test was assumed to have 100% reduction, a NO_x_ reduction efficiency of 90% or higher could be obtained under the conditions that exceed Section 1, as shown in Figure 6. The NO_x_ reduction efficiency was reduced to approximately 50% under the conditions between Section 1 and Section 2. Under the conditions of Section 3, the NO_x_ reduction efficiency sharply decreased compared with those of Section 1 and Section 2. This confirms that it was reduced to less than 40% compared with those achieved under the ISO standard conditions.

Thus, it was confirmed that the NO_x_ reduction efficiency is less than 40% compared with those achieved under the ISO standard conditions that can be obtained if the TiO_2_ photocatalysts are applied to vertical surfaces. In this case, the UV-A irradiance is reduced owing to the influence of reflected light other than the roofs where the UV rays reach directly. It was also confirmed that a similar NO_x_ reduction effect can be obtained in winter when the solar altitude is low or on days when the cloud coverage is high.

## 5. Conclusions

The purpose of this study is to reduce the concentration of PM precursors. In this study, the NO_x_ concentration reduction performance of TiO_2_ photocatalysts through their reactions with UV-A was analyzed as a method for reducing NO_x_ in secondary sources. The findings of this study can be summarized as follows.

First, the conditional test results that meet the international standard test standard of TiO_2_ photocatalyst performance evaluation showed that the TiO_2_ coating material used in this study had a NO_x_ reduction effect. The presence of Ti and O in the coating agent was confirmed through FESEM and EDS analysis.

Second, the NO_x_ reduction effect of TiO_2_ coating material was confirmed through changes in UV-A irradiance and NO concentration. The UV-A irradiance and concentration were reduced to 25%, 50%, and 75% compared to the ISO standard experimental method, and the NO_x_ reduction effect was also changed. It is expected that the NO_x_ reduction effect can be obtained even in the actual NO_x_ concentration and the UV-A irradiance in the atmosphere.

Third, according to the changed UV-A irradiance and NO concentration, it was confirmed that the factor that significantly affects NO_x_ reduction is the NO concentration rather than the UV-A irradiance. In addition, when UV-A irradiance is 7.5 W/m^2^ and NO concentration is 0.75 ppm or more, the reduction effect is confirmed to increase significantly.

Fourth, it was discovered that the NO_x_ reduction efficiency is less than 40% compared with those achieved under the ISO standard conditions that can be obtained if the TiO_2_ photocatalysts are applied to vertical surfaces where the UV-A irradiance is reduced. It was also confirmed that a similar NO_x_ reduction effect can be obtained in winter when the solar altitude is low compared with in summer or days when the cloud coverage is high.

Therefore, it is expected that the coating material mixed with TiO_2_ photocatalysts used in this study can be applied to existing buildings and structures. In addition, it is also highly applicable to building materials in which the direct application of TiO_2_ photocatalysts is difficult. It was also confirmed that the coating material can be used to reduce PM precursors because it can reduce the NO_x_ concentration in atmospheric environments. Therefore, the findings of this study are expected to be used to evaluate the performance of various building materials that are mixed with TiO_2_ photocatalysts in the future.

## Figures and Tables

**Table 1:** TiO_2_ coating composition.

Contained Chemicals	Proportion
Titanium dioxide (anatase)	1.75%
Silicone compound	5.6%
Ethanol	41.6%
Water	51.0%
Other	0.05%

**Table 2:** Field emission scanning electronic microscope (FESEM) and energy dispersive spectroscopy (EDS) measuring equipment.

Classification	Contents
Model	SIGMA 500 (Carl Ziess)
Detector	SE 2
EDS detector	X-Max^N^50 (Oxford)
Acceleration voltage	18.0 kV
Working distance	8.5 mm
Magnification	500× to 1000×
Time-resolution	0.8 nm

**Table 3:** STUB (Sample Holder) composition.

Element	wt%
C	12.84
O	3.04
Al	79.34
Cu	4.79
Total	100.00

**Table 4:** TiO_2_ coating composition.

Element	wt%
C	3.47
O	65.09
Al	10.39
Ti	21.05
Total	100.00

**Table 5:** Specifications of measuring equipment.

**Classification**	**AMI 310, SOM 900(KIMO, Montpon, France)**			
	**Measurement Range**	**Accuracy**	**Resolution**	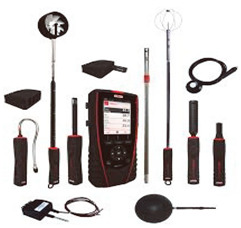
Temperature	−20 to +80 °C	±3% of the leading value ± 0.25 °C	0.1 °C	
Relative humidity	0–100% RH	Accuracy: ±1.8% RH Calibration Uncertainty: ±0.88% RH	0.1% RH	
Velocity	0.00–5.00 m/s	±3% of the leading value ± 0.05 m/s	0.01 m/s	
**Classification**	**Serinus 40 (ECOTECH, Melbourne, Austraila)**			
Range	Automatic	0–20 ppm		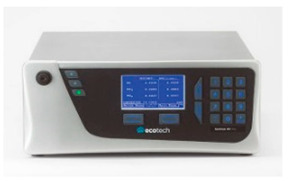
	USEPA approval	0.0–0.5 ppm		
	TUVEN certified	Less than NO (0–1000 ppm), NO_2_ (0–260 ppm)		
Accuracy/precision	Precision	0.4 ppb or 0.5% of reading (the lesser of the two)		
	Linearity	±1% of the total scale		
	Reaction time	90% in 15 s		
	Sample flow rate	0.3 slpm (total flow rate of 0.6 slpm for the NO and NO_x_ flow path)		

**Table 6:** ISO standard condition test values.

Classification	Value
UV-A irradiance	10 W/m^2^
NO gas concentration	1.00 ppm
Temperature	25 ± 2.5 ℃
Relative humidity	50%
Test time/measurement interval	3 h/1 min
Number of experiments	3 times

**Table 7:** Changes in condition test value.

Classification	UV-A Irradiance (W/m^2^)							
	2.50		5.00		7.50		10.0	
**NO concentration (ppm)**	CASE 1	0.25	CASE 5	0.25	CASE 9	0.25	CASE 13	0.25
	CASE 2	0.50	CASE 6	0.50	CASE 10	0.50	CASE 14	0.50
	CASE 3	0.75	CASE 7	0.75	CASE 11	0.75	CASE 15	0.75
	CASE 4	1.00	CASE 8	1.00	CASE 12	1.00	CASE 16	1.00

**Table 8:** ISO standard condition test result.

Classification	Start Concentration	End Concentration	Reduction Rate (Reduction Amount)
**NO**	1.015 ppm	0.653 ppm	35.67% (12.06 µmol/10 cm^2^·3 h)
**NO_2_**	0.000 ppm	0.143 ppm	3.11 µmol/10 cm^2^·3 h (generated)
**NO_x_**	1.015 ppm	0.796 ppm	21.58% (8.95 µmol/10 cm^2^·3 h)

**Table 9:** Test for changes in the condition that resulted in NO reduction.

	**NO Concentration (ppm)**	**Reduction in Concentration (a)**		
**UV-A Irradiance (W/m^2^)**		0.25 ppm	0.50 ppm	0.75 ppm
2.50 W/m^2^		0.119 ppm	0.179 ppm	0.189 ppm
5.00 W/m^2^		0.108 ppm	0.136 ppm	0.234 ppm
7.50 W/m^2^		0.113 ppm	0.201 ppm	0.327 ppm

**Table 10:** NO_2_ generation results based on NO concentration and UV-A irradiance.

	**NO Concentration (ppm)**	**Increase in Concentration (b)**		
**UV-A Irradiance (W/m^2^)**		0.25 ppm	0.50 ppm	0.75 ppm
2.50 W/m^2^		0.053 ppm	0.105 ppm	0.091 ppm
5.00 W/m^2^		0.028 ppm	0.060 ppm	0.088 ppm
7.50 W/m^2^		0.024 ppm	0.076 ppm	0.094 ppm

**Table 11:** Test results of changes in condition to achieve NO_x_ reduction.

	**NO Concentration (ppm)**	**NO_x_ Reduction Concentration (c = a − b)**		
**UV-A Irradiance (W/m^2^)**		0.25 ppm	0.50 ppm	0.75 ppm
2.50 W/m^2^		0.067 ppm	0.074 ppm	0.098 ppm
5.00 W/m^2^		0.080 ppm	0.076 ppm	0.121 ppm
7.50 W/m^2^		0.089 ppm	0.126 ppm	0.202 ppm

**Table 12:** NO_x_ reduction amount.

	**NO Concentration (ppm)**	0.25	0.50	0.75	1.00
**UV-A Irradiance (W/m^2^)**		**Reduction Amount (µmol/10 cm^2^·3 h)**			
2.50		**2.81**	**3.68**	4.32	5.41
5.00		2.99	3.23	5.34	5.45
7.50		3.24	5.05	8.18	7.99
10.0		4.45	6.08	8.07	8.95
